# Supplementary material for: Driven translocation of a semiflexible polymer through a conical channel in the presence of attractive surface interactions
Source: Sci Rep. 2022 Nov 9;12:19081. doi: 10.1038/s41598-022-21845-6 (PMC9646819; doi:10.1038/s41598-022-21845-6)
Supplement: Supplementary file 4 — Supplementary Information. [file 41598_2022_21845_MOESM4_ESM.pdf]

I. DESCRIPTION OF THE SUPPLEMENTARY VIDEOS

The three videos under the supplementary section are attached to better convey the dynamics of the translocation process.

A. Construction of the system and color code (same for all the three (*.mp4) videos)

- The polymer is made up of 64 solid spherical beads which are red in color. .
- The static conical pore is made up of 16 solid spherical beads which are blue in color.
- The walls are constructed at the narrow and wide openings of the pore along the y- direction, and are shown in green color.

B. Details for the specific videos

- Apex5-Kappa8.mp4: This video shows the translocation process of a polymer with $\kappa = 8$ through a conical pore with $\alpha = 5^\circ$ and $f_0 = 0.2$. The video starts once the first bead of the equilibrated polymer enters the pore from the narrow opening of the pore (*cis* side) and ends when all the beads of the polymer have exited from the wider opening of the conical pore (*trans* side).
- Apex10-Kappa8.mp4: This video shows the translocation process of a polymer with $\kappa = 8$ through a conical pore with $\alpha = 10^\circ$ and $f_0 = 0.2$. The video starts once the first bead of the equilibrated polymer enters the pore from the narrow opening of the pore (*cis* side) and ends when all the beads of the polymer have exited from the wider opening of the conical pore (*trans* side).
- Apex15-Kappa8.mp4: This video shows the translocation process of a polymer with $\kappa = 8$ through a conical pore with $\alpha = 15^\circ$ and $f_0 = 0.2$. The video starts once the first bead of the equilibrated polymer enters the pore from the narrow opening of the pore (*cis* side) and ends when all the beads of the polymer have exited from the wider opening of the conical pore (*trans* side).
